# Facile Synthesis of Coaxial CNTs/MnO_x_-Carbon Hybrid Nanofibers and Their Greatly Enhanced Lithium Storage Performance

**DOI:** 10.1038/srep17473

**Published:** 2015-12-01

**Authors:** Zunxian Yang, Jun Lv, Haidong Pang, Wenhuan Yan, Kun Qian, Tailiang Guo, Zaiping Guo

**Affiliations:** 1 National & Local United Engineering Laboratory of Flat Panel Display Technology, Fuzhou University, Fuzhou 350002, P. R. China; 2 Institute for Superconducting & Electronic Materials, University of Wollongong, NSW 2522, Australia; 3 School of Mechanical, Materials & Mechatronics Engineering, University of Wollongong, NSW 2522, Australia

## Abstract

Carbon nanotubes (CNTs)/MnO_x_-Carbon hybrid nanofibers have been successfully synthesized by the combination of a liquid chemical redox reaction (LCRR) and a subsequent carbonization heat treatment. The nanostructures exhibit a unique one-dimensional core/shell architecture, with one-dimensional CNTs encapsulated inside and a MnO_x_-carbon composite nanoparticle layer on the outside. The particular porous characteristics with many meso/micro holes/pores, the highly conductive one-dimensional CNT core, as well as the encapsulating carbon matrix on the outside of the MnO_x_ nanoparticles, lead to excellent electrochemical performance of the electrode. The CNTs/MnO_x_-Carbon hybrid nanofibers exhibit a high initial reversible capacity of 762.9 mAhg^−1^, a high reversible specific capacity of 560.5 mAhg^−1^ after 100 cycles, and excellent cycling stability and rate capability, with specific capacity of 396.2 mAhg^−1^ when cycled at the current density of 1000 mAg^−1^, indicating that the CNTs/MnO_x_-Carbon hybrid nanofibers are a promising anode candidate for Li-ion batteries.

With the popularization of mobile electronic devices, lithium ion batteries (**LIBs**), as inexpensive, flexible, lightweight, and environmentally friendly energy storage device, have attracted more and more attention due to their high output voltage and high energy density^1^,^2^,^3^,^4^,^5^,^6^,^7^. Many electrode materials, especially anode materials, such as CuO^8^,^9^,^10^, SnO_2_^11^,^12^,^13^, TiO_2_^14^,^15^,^16^,^17^,^18^, and some other transition metal oxides, have been investigated and used for the lithium ion battery. Of all those anode materials mentioned-above, manganese dioxide has been of great interest to many researchers on the lithium ion battery, mainly due to its particular advantages of high storage capacity, low cost, lower thermodynamic equilibrium voltage versus Li/Li^+^, and environmental friendliness^19^,^20^,^21^. The practical implementation of manganese oxide in lithium-ion batteries, however, is greatly hindered by its poor cycling performance owing to its poor conductivity, as well as its vulnerability to agglomeration and mechanical strain arising from the large volume variation during the lithium charge/discharge processes, which results in increased diffusion lengths and serious electrical disconnection^20^,^22^.

Recently, many efforts have been made to solve the problems mentioned above. One effective strategy is to reduce the particle size of manganese oxide down to the nanometer scale, which could accommodate the volume changes, and furthermore, offer more active sites for lithium ions during charge/discharge cycling^23^,^24^,^25^, with these advantages mainly due to the higher specific surface area of the nanoparticles. Many nanosize forms of manganese oxide, such as nanotubes^23^, nanobelts^26^, nanofibers^27^, nanospheres^28^,^29^, nanorods^30^, and so on, have been prepared by many different methods, including the hydrothermal method^26^, the solvothermal route^31^, the electrospinning method^32^, electrochemical techniques, etc. Another alternative strategy is to disperse manganese oxide in the form of nanoparticles into a matrix with good conductivity, which could cushion the mechanical effects stemming from the volume changes during the charge/discharge process and simultaneously improve the conductivity of the composite^33^,^34^. Generally, the carbonaceous materials have been the most promising candidates because of their good conductivity and chemical compatibility with manganese oxide. Many carbonaceous materials, such as carbon nanotubes^24^, carbon nanofibers^35^,^36^, and graphene^37^, have been used to fabricate carbon/manganese oxide nanocomposites as anode materials for lithium ion batteries. These carbon/manganese oxide nanocomposites, however, are still a long way from being ideal anode candidates for the lithium ion battery, mainly because there are many manganese oxide nanoparticles outside the composite that can come into direct contact with each other, which also results in some polarization effects and large volume variation during lithium charge/discharge processes. So, there are still many challenges involved in solving the problems of volume variation and poor conductivity mentioned above.

In this paper, a simple and low-cost approach is reported to prepare coaxial carbon nanotubes (CNTs)/MnO_x_-Carbon hybrid nanofibers by a liquid chemical redox reaction (LCRR), followed by a subsequent carbonization treatment. In addition to combining the advantages of the nanoscale manganese oxide particles with those of the carbon matrix, the unique CNTs/MnO_x_-Carbon hybrid nanofibers have more unique advantages, such as one-dimensional nanostructures, meso/micro porosities, large surface-to-volume ratio, and excellent conductivity, including both ionic conductivity for lithium ions and electronic conductivity, mainly owing to the highly conductive CNTs on the inside and the carbon on the outside of the MnO_x_ nanoparticles. The CNTs/MnO_x_-Carbon hybrid nanofibers have been investigated in a preliminary way for potential use as an anode material for the lithium ion battery and have exhibited excellent cycling stability and rate capability.

## Results

**Figure 1:**
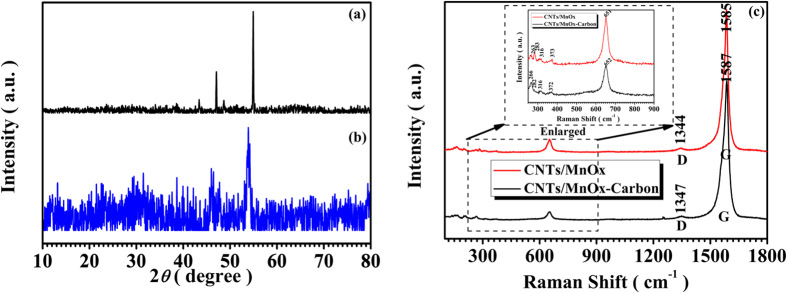
X-ray diffraction patterns and Raman spectra of as-prepared CNTs/MnO_x_ and CNTs/MnO_x_-Carbon hybrid nanofibers: (**a**) XRD CNTs/MnO_x_ and (**b**) XRD of CNTs/MnO_x_-Carbon hybrid nanofibers with different valences of manganese oxide (Mn_3_O_4_: Hausmannite structure, JCPDS 75–1560; Mn_2_O_3_: Bixbyite structure, JCPDS 71–0636; MnO: Manganosite structure, JCPDS 72–1533), as indexed in the patterns; (**c**) Raman spectra of the CNTs/MnOx and CNTs/MnOx-Carbon hybrid nanofibers, respectively.

As illustrated in Supporting Information Fig. S1 and Fig. 1(a), the X-ray diffraction patterns of the as-prepared MnO_2_, CNTs/MnO_x_, and CNTs/MnO_x_-Carbon hybrid nanofibers reveal that the precursors, the manganese oxide nanoparticles, are α-MnO_2_ with a tetragonal structure (JCPDS 72–1982) (see Fig. S1) and undergo a subsequent phase transition into different valences of manganese oxide, possibly owing to the reducing reaction due to contact with the carbon matrix and CNTs. The manganese oxide in the CNTs/MnO_x_ hybrid nanofibers includes the main phase Mn_3_O_4_ with a hausmannite structure (JCPDS 75–1560) and the minor phase Mn_2_O_3_ with a bixbyite structure (JCPDS 71–0636) due to the reducing reaction from contact with the CNTs during the carbonization treatment at 500 °C, which is similar to the manganese oxide in CNTs/MnO_x_-Carbon hybrid nanofibers, except that there is some trace MnO phase in the CNTs/MnO_x_-Carbon hybrid nanofibers owing to more chances for the manganese oxide nanoparticles to come into contact with the carbon matrix in the composite, according to the intensity of their own X-ray diffraction (XRD) characteristic peaks. The Raman spectra of the CNTs/MnO_x_ and CNTs/MnO_x_-Carbon hybrid nanofibers in 100–1800 cm^−1^ region are acquired (see Fig. 1(b)). The obvious spectral feature in CNTs/MnO_x_ and CNTs/MnO_x_-Carbon hybrid nanofibers, where there are the strongest peak around the 1585 cm^−1^, named as “G” peak, and a very weak peak around the 1345 cm^−1^, named as “D” peak, possibly originating from the CNTs core in the two composites, belongs to the Raman characteristic of the carbon composite^38^. Additionally, the CNTs/MnO_x_ and CNTs/MnO_x_-Carbon hybrid nanofibers also exhibit a common spectral feature for all the all manganese oxides, where a relatively stronger phonon band in the 640−660 cm^−1^ region and a few weak phonon bands in the range from 200 to 480 cm^−1^ were found^39^,^40^. The phonon band with large scattering intensity in the range from 640−660 cm^−1^ were assigned to A_1g_ spectroscopic species with symmetric vibrations ν_2_(Mn−O) while the weak bands at about 370−200 cm^−1^ to Mn−O bending vibrations. Most of the vibrations found in these spectra were related to the motion of the oxygen atoms within the MnO_6_ octahedral units in all kinds of manganese oxides^39^ including Mn_3_O_4_, Mn_2_O_3_, MnO and so on, which is in well agreement with the XRD result as described above. Here, the liquid chemical redox reaction (LCRR) at first result in the formation of the special architecture with the CNTs core and the MnO_2_ nanoparticles shell covered with a thin layer of PVP polymer around them. And the subsequent carbonization treatment processes finally facilitate the conversion from the parent pure α-MnO_2_ phase into the composite phases of manganese oxide, mainly owing to the reducing reaction between the MnO_2_ and the carbon matrix at the high temperature^36^,^41^,^42^,^43^, and simultaneously, the carbon shell formation outside of the MnO_x_ nanoparticles originating from the PVP polymer layer covered outside before. These CNTs/MnO_x_-Carbon hybrid nanofibers, consisting of CNT cores and low-valence manganese oxide-carbon composite shells with excellent conductivity, effectively inherit the one-dimensional structure of the CNTs, which may enhance the conductivity of the hybrid nanofibers, mainly due to their particular architecture with one-dimensional structure, the CNT cores, and the highly conductive carbon matrix coated outside.

**Figure 2:**
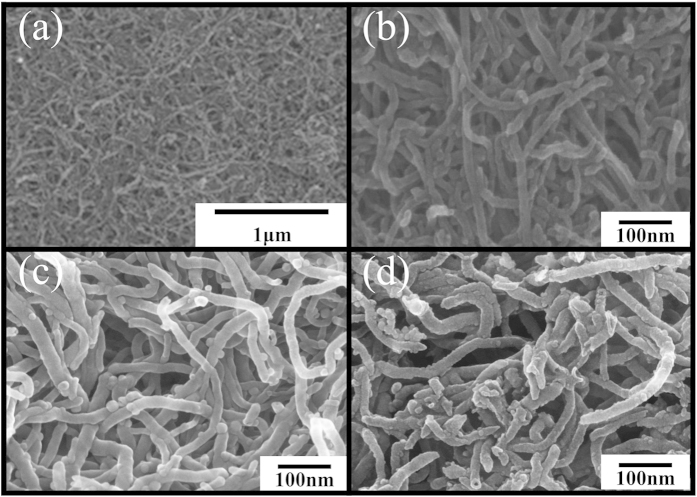
FE-SEM images of as-prepared CNTs/MnO_x_ and CNTs/MnO_x_-Carbon hybrid nanofibers: (**a**) acidized CNTs, (**b**) high magnification image of the acidized CNTs, (**c**) CNTs/MnO_x_, (**d**) CNTs/MnO_x_-Carbon hybrid nanofibers.

The morphologies of the as-prepared MnO_2_ powders, CNTs/MnO_x_, and CNTs/MnO_x_-Carbon hybrid nanofibers, as well as the acidized CNTs, have been respectively investigated by field-emission scanning electron microscopy (FE-SEM) (see Fig. 2 and Supporting Information Fig. S2). The pure MnO_2_ nanoparticles prepared by LCRR with diameters of only a few nanometers (see Fig. S2) are so small that only some aggregates consisting of many tiny MnO_2_ nanoparticles can be seen. As shown in Fig. 2, the acidized CNTs, the one-dimensional template precursors, with diameters of 15–20 nm and lengths extending to a few micrometers are randomly aligned. After the liquid chemical redox reaction (LCRR) and subsequent carbonization treatment in argon at 500 °C for 2 h, the one-dimensional morphology is maintained (see Fig. 2(c,d)), and the CNTs/MnO_x_-PVP hybrid nanofibers have finally been converted to fully carbonized CNTs/MnO_x_-Carbon hybrid nanofibers with diameters of 20–30 nm, which are slightly thicker than those of the acidized CNTs. Both the CNTs/MnO_x_ hybrid nanofibers and the CNTs/MnO_x_-Carbon hybrid nanofibers are encapsulated by many MnO_x_ nanocrystals than 5 nm in size, which are tightly aligned around the CNT core, and those MnO_x_ nanoscale particles are too small to be discerned clearly from each other. Additionally, the template precursor, the acidized CNTs, the as-prepared CNTs/MnO_x_-Carbon hybrid nanofibers, and the CNTs/MnO_x_ hybrid nanofibers all have become attached together to form many meso/micro holes/voids/pores (see Fig. 2(b–d)). These meso/micro holes/voids/pores that are formed in these hybrid nanofibers, as well as the high content of carbon matrix (see Supporting Information Fig. S3) with high conductivity, including both the CNT cores and the carbon matrix coated on the outside, would ensure a high electrode-electrolyte contact area, so that a large amount of lithium ions can be accommodated without any remarkable degradation of the structure during the charge/discharge cycling, which is favorable for both lithium ion storage and lithium ion diffusion.

**Figure 3:**
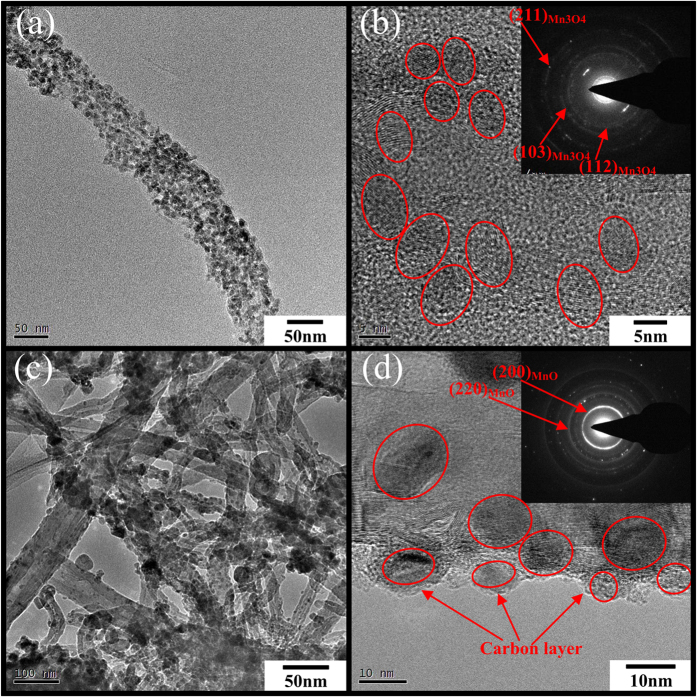
(**a**) Low-magnification TEM image of CNTs/MnO_x_ hybrid nanofibers, (**b**) HRTEM image and SAED pattern (inset) of a section of CNTs/MnO_x_ hybrid nanofiber; (**c**) low-magnification TEM image of CNTs/MnO_x_-Carbon hybrid nanofibers, (**d**) HRTEM image and SAED pattern (inset) of a section of CNTs/MnO_x_-Carbon hybrid nanofiber.

Transmission electron microscopy (TEM) of the as-synthesized MnO_2_ powders, and the CNTs/MnO_x_ and CNTs/MnO_x_-Carbon hybrid nanofibers has shed further light on their structural and morphological characteristics (see Fig. 3 and Supporting Information Fig. S4). As shown in Fig. 3(a), many tiny MnO_x_ nanoparticles with diameters of ~5 nm, inherited from the tiny nature of the MnO_2_ nanoparticle precursor (see Fig. S4), are uniformly and tightly loaded on the surfaces of the CNTs to form a core/shell architecture, which is in good accordance with the FE-SEM results (see Fig. 2(c)). As illustrated in Fig. 3(b), the high resolution transmission electron microscope (HRTEM) image of the CNTs/MnO_x_ nanofibers, as well as the corresponding selected area electronic diffraction (SAED) pattern (see Fig. 3(b) inset), reveals that the loading of the manganese oxide particles on the outside of the CNTs, consisting of many manganese oxide nanocrystals, ~5 nm in diameter, is probably attributable to their polycrystalline nature. In Fig. 3(c,d), in the case of the CNTs/MnO_x_-Carbon hybrid nanofibers, there is an obvious amorphous carbon layer coated on the outside of the MnO_x_ nanoparticles, with thickness of 1–2 nm. As shown in Fig. 3(d), the high resolution transmission electron microscope (HRTEM) image and selected area electronic diffraction (SAED) pattern of the CNTs/MnO_x_-Carbon hybrid nanofibers further demonstrate that the MnO_x_ in the CNTs/MnO_x_-Carbon hybrid nanofibers also consists of many nanoparticles/nanocrystals that are uniformly and tightly aligned on the surfaces of the CNTs to form a one-dimensional core/shell morphology, except that both the MnO_x_ nanocrystals and the amorphous carbon form the shell layer in the CNTs/MnO_x_-Carbon hybrid nanofibers. The SAED patterns of the CNTs/MnO_x_ and CNTs/MnO_x_-Carbon hybrid nanofibers are characterized by complex diffraction spots, which indicates that the MnO_x_ in these composites exists in polycrystalline form, owing to the reducing reaction between the MnO_2_ and the carbon matrix during the carbonization treatment, with the products mainly including Mn_3_O_4_, Mn_2_O_3_, and MnO nanocrystals (see Fig. 1), respectively, which is similar to what has been reported previously^36^,^42^. Additionally, the manganese oxide contents in the CNTs/MnO_x_ and CNTs/MnO_x_-Carbon hybrid nanofibers are ~56.6% and ~47.1%, respectively, according to the TGA results (see Fig. S3).

**Figure 4:**
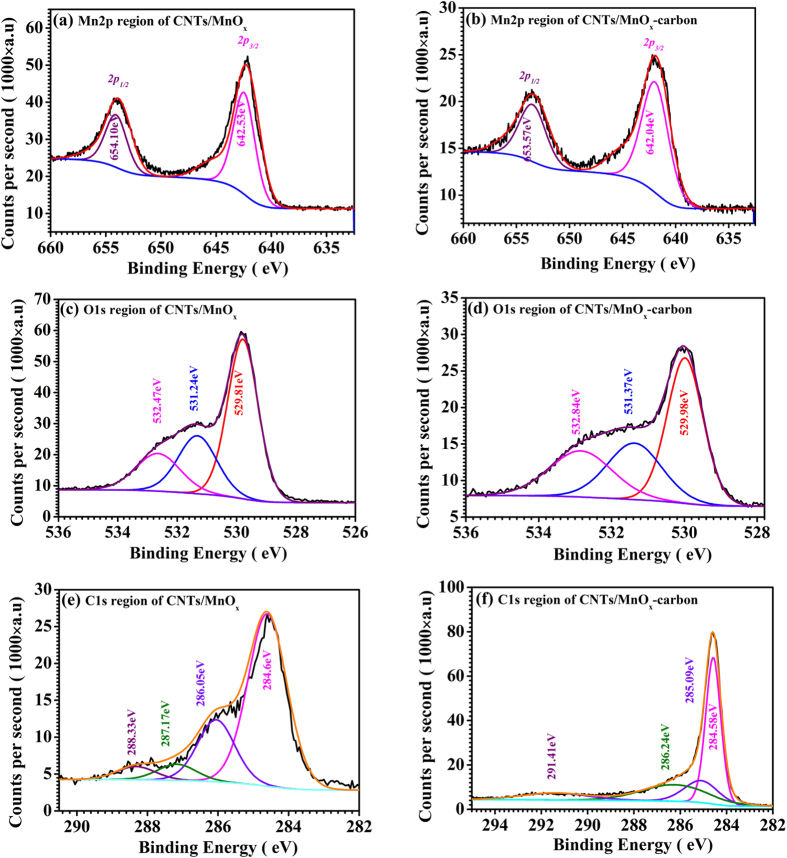
XPS high-resolution spectra of the Mn 2p, O1s, and C1s regions of the as-prepared CNTs/MnO_x_ and CNTs/MnO_x_-Carbon hybrid nanofibers. (**a**) Mn 2p region of CNTs/MnO_x_ nanofibers; (**b**) Mn2p region of CNTs/MnO_x_-Carbon nanofibers; (**c**) O1s region of CNTs/MnO_x_ nanofibers; **(d)** O1s region of CNTs/MnO_x_-Carbon nanofibers; (**e**) C1s region of CNTs/MnO_x_ nanofibers; (**f**) C1s region of CNTs/MnO_x_-carbon nanofibers.

X-ray photoelectron spectroscopy (XPS) of the MnO_2_ powders, and the CNTs/MnO_x_ and CNTs/MnO_x_-Carbon hybrid nanofibers was conducted from 0 to 1100 eV. Obvious Mn 2p, O1s, and C1s peaks for the CNTs/MnO_x_ and CNTs/MnO_x_-Carbon hybrid nanofibers were detected, and their high-resolution spectra are shown in Fig. 4(a–f), respectively. The Mn 2p spectrum (Fig. 4(a)) for the CNTs/MnO_x_ hybrid nanofibers comprises two symmetrical peaks with binding energies (BEs) at 642.53 eV and 654.10 eV, which are attributable to Mn 2p_3/2_ and Mn 2p_1/2_, respectively. The separation between these two peaks for the CNTs/MnO_x_ hybrid nanofibers is 11.57 eV, which is approximately equal to that for the CNTs/MnO_x_-Carbon hybrid nanofibers (see Fig. 4(b)), consisting of two symmetrical peaks with binding energies (BEs) at 642.04 eV and 653.57 eV, but the peaks are obviously larger than those for the pure MnO_2_ nanoparticles (see Fig. S5(a)), which possess two symmetrical peaks with binding energies (BEs) at 642.43 eV and 653.85 eV. The cause is possibly that there is some low valence manganese oxide that is present in both the CNTs/MnO_x_ and the CNTs/MnO_x_-Carbon hybrid nanofibers reduced by the carbonaceous materials at 500 °Cfor 2 h under argon atmosphere, which is in good accordance with previous reports^36^,^42^,^43^. As for the O1s spectrum in the CNTs/MnO_x_ composite (Fig. 4(c)), the CNTs/MnO_x_-Carbon composite (Fig. 4(d)), and the pure MnO_2_ nanoparticles (Fig. S5(b)), the main portion of the response could come from Mn-O bonds in the manganese oxide, as evidenced by the O1s binding energy (BE) peaks at ~529.81 eV (Fig. 4(c)), 529.98 eV (Fig. 4(d)), and 529.67 eV (Fig. S5(b)), while the peaks at 531.24 eV (Fig. 4(c)), 530.37 eV (Fig. 4(d)), and 531.13 eV (Fig. S5(b)) may be attributable to the OH^−^ radical, adsorbed oxygen, or carbonyl groups^44^, partly arising from the incomplete pyrolysis of the carbon-containing polymer (PVP) during the encapsulation with the carbon matrix. As for the high BE peaks at 532.47 eV (Fig. 4(c)), 532.84 eV (Fig. 4(d)), and 532.73 eV (Fig. S5(b)), they possibly originate from a small amount of absorbed H_2_O on the outside^44^. In addition, the use of CNTs, together with the encapsulation in the carbon matrix of the composite nanofibers, possibly leads to the slight reduction in the three fitted O1s peaks compared to the pure α-MnO_2_ nanoparticles. In the case of the C1s spectrum for the CNTs/MnO_x_ and CNTs/MnO_x_-Carbon composites (see Fig. 4(e,f)), the strongest peaks at 284.60 eV (Fig. 4(e)) and 284.58 eV (Fig. 4(e)) are attributed to the C-C bonds that exist in the CNTs or the encapsulating carbon matrix, while the following peaks at 286.05 eV (Fig. 4(e)) and 285.09 eV (Fig. 4(f)) are partly attributable to the presence of some oxygen-containing functional groups in the organic matrix after the heat treatment at relatively low temperature (500 °C), including some disordered carbon or oxidant carbon, such as carbon in alcohols^44^, which is in good accordance with the fitted O1s peaks mentioned above. The remaining two small peaks at 287.17 eV and 288.33 eV (Fig. 4(e)), and the corresponding peaks at 287.24 eV and 291.41 eV (Fig. 4(f)), possibly come from a trace amount of carboxyl in the hybrid samples^43^,^44^,^45^. From a combination of the XRD, FE-SEM, and TEM results, together with the XPS results, it is concluded that the CNTs/MnO_x_ and CNTs/MnO_x_-Carbon hybrid nanofibers, inheriting their one-dimensional morphology from the parent CNT templates, are composed of CNT cores with MnO_x_ or MnO_x_-Carbon shells, in the form of main phase Mn_3_O_4_ and some minor phases of Mn_2_O_3_ and MnO, with or without carbon. These one-dimensional CNTs/MnO_x_-Carbon hybrid nanofibers possess many unique advantages in lithium ion battery application^36^,^41^,^42^,^43^, such as high conductivity, owing to encapsulation of the manganese oxide nanoparticles in the highly conductive carbon matrix, improved Li^+^ and electrolyte transport in the hybrid nanomaterials because of the micron-size holes/voids/pores formed between the bound/attached CNTs/MnO_x_-Carbon nanofibers, etc., all of which would favor greatly enhanced electrochemical performance of the electrode as compared with the pure MnO_2_ nanoparticles and even the CNTs/MnO_x_ hybrid nanofiber electrodes.

**Figure 5:**
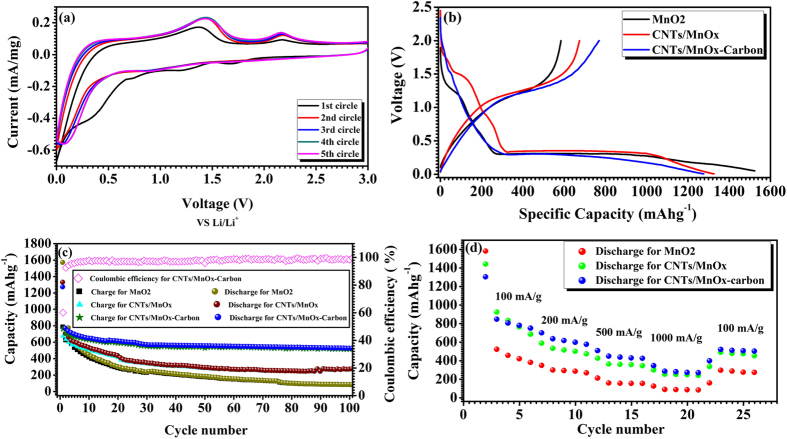
Electrochemical performances of MnO_2_ nanoparticle, CNTs/MnO_x_ hybrid nanomaterial, and CNTs/MnO_x_-Carbon hybrid nanofiber electrodes cycled between 0.01 and 3.0 V vs. Li^+^/Li: (**a**) Cyclic voltammograms of CNTs/MnO_x_-Carbon hybrid nanomaterial electrode of the first 5 cycles at a scan rate of 0.1 mVs^−1^ in the voltage range of 0.01–3.0 V. (**b**) Voltage profiles for the first cycle of the MnO_2_ nanoparticle, CNTs/MnO_x_ hybrid nanofiber, and CNTs/MnO_x_-Carbon hybrid nanofiber electrodes at the current density of 100 mAg^−1^. (**c**) Capacity vs. cycle number curves and coulombic efficiency from the first cycle to the 101^st^ cycle for the MnO_2_ nanoparticle, CNTs/MnO_x_ hybrid nanofiber and CNTs/MnO_x_-Carbon hybrid nanofiber electrodes at the current density of 100 mAg^−1^, with cutoff voltage betwen 0.01 and 3.0 V. (**d**) Rate capabilities of MnO_2_ nanoparticle, CNTs/MnO_x_ hybrid nanofiber, and CNTs/MnO_x_-Carbon hybrid nanofiber electrodes at various current densities (100 mAg^−1^, 200 mAg^−1^, 500 mAg^−1^, 1000 mAg^−1^).

The electrochemical performances of the MnO_2_ nanoparticles, and the CNTs/MnO_x_ and CNTs/MnO_x_-Carbon hybrid nanofibers electrodes, including in galvanostatic discharge-charge cycling and cyclic voltammetry, have been systematically investigated (Fig. 5 and Supporting Information Fig. S6(a)). Cyclic voltammograms (CVs) of the CNTs/MnO_x_-Carbon hybrid nanofibers at a scan rate of 0.1 mVs^−1^ from the 1^st^ cycle to the 5^th^ cycle, with the cut-off voltage window between 0.01 and 3.0 V, are presented in Fig. 5(a), while those of the CNTs/MnO_x_ hybrid nanofibers, including the 1^st^, 2^nd^, and 5^th^ cycles, are presented in Fig. S6(a). The curve of the first cycle is obviously different from those of the later ones, which is possibly attributable to the formation of an inactive solid electrolyte interphase (SEI)^36^,^42^ at the first cycle. From the 2^nd^ cycle onward, highly reversible CV curves are obtained. From the 2^nd^ cycle on, there is an obvious anodic peak at about 1.43 V vs. Li/Li^+^ and a broad cathodic peak at 0.89 V. The two peaks are probably attributable to the reversible oxidation/reduction between manganese oxide and lithium^41^. Whereas, the cathodic/anodic peak pair at 0.06 V and 0.349 V is possibly attributable to the lithium ion insertion into/extraction out of the carbon matrix. Noticeably, there is one unknown peak at 2.17 V in all the cycles, which needs further investigation.

The cycling performances of the MnO_2_ nanoparticles, and the CNTs/MnO_x_ and CNTs/MnO_x_-Carbon hybrid nanofiber electrodes were further explored in the voltage range of 3.0–0.01 V (vs Li/Li^+^) at a constant current density of approximately 100 mAg^−1^ up to 100 cycles. Figure 5(b) presents the voltage profiles of the MnO_2_ nanoparticle, and the CNTs/MnO_x_ and CNTs/MnO_x_-Carbon hybrid nanofiber electrodes at the current density of 100 mAg^−1^. The first discharge and charge steps of the CNTs/MnO_x_-Carbon hybrid nanofiber electrode deliver a specific capacity of 1275.9 and 762.9 mAhg^−1^, respectively, while the CNTs/MnO_x_ nanofiber electrode and the pure MnO_2_ nanoparticle electrode deliver 1326.0 and 674.2 mAhg^−1^, and 1726.3 and 847.8 mAhg^−1^ in the first discharge and charge steps, respectively. The initial coulombic efficiency of the CNTs/MnO_x_-Carbon composite electrode is above 60.3%, very much higher than those of the CNTs/MnO_x_ composite (50.5%) and the pure MnO_2_ electrode (49.1%), which is possibly attributable to the encapsulation by the highly conductive carbon matrix on the outside for the CNTs/MnO_x_-Carbon composite^36^,^42^ and also its particular one-dimensional core/shell architecture. Figure 5(c) shows the discharge/charge capacity versus cycle number for the MnO_2_ nanoparticle electrode, and the CNTs/MnO_x_ and CNTs/MnO_x_-Carbon composite electrodes at the current density of 100 mAg^−1^, respectively. The CNTs/MnO_x_-Carbon composite electrode exhibits excellent cycling performance and a high reversible specific capacity of over 662 mAhg^−1^ after the first 10 cycles. Moreover, it retains a high reversible specific capacity of 560.5 mAhg^−1^ after 100 cycles, with high coulombic efficiency of nearly 100%, which is not only much higher than the specific capacity of the pure MnO_2_ electrode (189.5 mAhg^−1^), but also obviously higher than that of the CNTs@MnO_x_ electrode (401.5 mAhg^−1^) after 100 cycles. As indicated in Fig. 5(d), the CNTs/MnO_x_-Carbon composite electrode exhibits excellent rate performance. It also delivers a discharge capacity of over 840 mAhg^−1^ at the current density of 100 mAg^−1^, 632.9 mAhg^−1^ at 200 mAg^−1^, 513.2 mAhg^−1^ at 500 mAg^−1^, and 346.1 mAhg^−1^ at 1000 mAg^−1^, respectively, and finally recovers to around 529.3 mAhg^−1^ when the current density goes back to 100 mAg^−1^, which is much better than the performances of the pure α-MnO_2_ nanoparticle electrode and the CNTs/MnO_x_ hybrid nanofiber electrode. Therefore, the great enhancement of the initial capacity, coulombic efficiency, reversible discharge capacity, and rate capability for the CNTs/MnO_x_-Carbon composite electrode possibly should be ascribed to the short Li^+^ diffusion paths and the easy access of the electrolyte to the active material through the interconnected meso-/micro-pores formed by the irregularly bonded composite nanofibers (see Fig. 2), as well as the good electrical connectivity owing to the encapsulation by the carbon matrix on the outside (see Fig. 3 and Fig. S6(b)).

## Discussion

The formation and the charge diffusion mechanism of the CNTs/MnO_x_-Carbon nanofibers are illustrated in Scheme S1 (Supporting Information). During liquid chemical redox reaction (LCRR), the special architecture formed with the CNTs core and the MnO_2_ nanoparticles shell covered with a thin layer of PVP polymer. And after the subsequent carbonization treatment processes, the parent pure α-MnO_2_ phase would be converted into the composite phases of manganese oxide, mainly owing to the reducing reaction between the MnO_2_ and the carbon matrix under the high temperature condition^36^,^41^,^42^,^43^, and simultaneously, the PVP polymer layer covered outside be carbonized into the carbon shell outside of the MnO_x_ nanoparticles. The numerous MnO_x_ nanoparticles encapsulated in the carbon matrix are uniformly dispersed on the outside of the CNTs and form the one-dimensional morphology with core/shell architecture. Many meso/micro holes/pores have been formed by those irregularly-bonded CNTS/MnO_x_-Carbon nanofibers. Although the CNTs/MnO_x_ composite might also inherit the morphology of the CNTs (Scheme S1(a)) and be characterized by one-dimensional architecture (Scheme S1(b)), there are more chances for the MnO_x_ nanoparticles in this composite to come into contact each other, which, together with the relatively poor conductivity, would finally lead to its relatively poor electrochemical performance. Accordingly, the particular one-dimensional architecture with the CNT cores and MnO_x_-Carbon composite shells, and the porous morphology with many meso/micro holes/pores formed by those irregularly-bonded CNTs/MnO_x_-Carbon nanofibers, as well as the relatively high specific surface area, all ensure good electrode-electrolyte contact and short lithium ion diffusion pathways during the discharge/charge cycling, and thus greatly enhance the lithium storage capacity and rate capability. Additionally, the carbon matrix coated on the outside of the MnO_x_ nanoparticles in the CNTs/MnO_x_-Carbon nanofibers, as well as the CNT cores, would greatly enhance the conductivity of the active material during the lithium intercalation/de-intercalation, which plays an essential role in the excellent lithium storage capacity, cyclability, and rate capability of this electrode.

In summary, CNTs/MnO_x_-Carbon hybrid nanofibers, each consisting of a CNT core and a MnO_x_-carbon composite shell, have been successfully synthesized by a facile liquid chemical redox reaction (LCRR), followed by a subsequent carbonization treatment in argon. As a potential anode material for lithium ion batteries, CNTs/MnO_x_-Carbon hybrid nanofibers exhibit a high initial reversible capacity of 762.9 mAhg^−1^ with coulombic efficiency of approximately 60.3% at the current density of 100 mAg^−1^, and further maintain a high reversible specific capacity of 560.5 mAhg^−1^ after 100 cycles with high coulombic efficiency of nearly 100%. The hybrid nanomaterials also exhibit good rate performance with specific capacity of 396.2 mAhg^−1^ when cycled at the current density of 1000 mAg^−1^. This particular one-dimensional core/shell architecture is characterized by the presence of many meso/micro holes/pores formed by the CNTs/MnO_x_-Carbon nanofibers. Those meso/micro holes/pores facilitate the lithium ion and electrolyte diffusion in these active materials during the charge/discharge processes. Furthermore, the CNT cores in the composite nanofibers, together with the carbon matrix outside of the MnO_x_ nanoparticles, would play an essential role in supporting the MnO_x_ nanoparticles, buffering the volume variation of the MnO_x_ nanoparticles during discharge/charge cycling, and greatly enhancing the conductivity of the active materials, as well as lithium ion and electrolyte diffusion. Therefore, the hybrid nanomaterials will be a very promising candidate as a potential anode material for LIBs, even though the composition and structure of those materials require further improvement.

## Methods

### Synthesis of CNTs/MnO_x_-Carbon hybrid nanofibers

CNTs/MnO_x_-Carbon hybrid nanofibers have been prepared by a liquid chemical redox reaction (LCRR), followed by a subsequent carbonization treatment. Firstly, the CNTs were treated with nitric acid at 110 °C for 3 h in order to remove the iron catalyst from the CNTs. Subsequently, 3 g of the acid-treated CNTs, 0.01 mol Mn(CHCOO)_2_·4H_2_O (Analytical Pure, Sinopharm Chemical Reagent Co. Ltd, China), and 0.0066 mol KMnO_4_ (Analytical Pure, Sinopharm Chemical Reagent Co. Ltd, China), as well as 0.0002 mmol polyvinyl pyrrolidone (PVP, MW = 1,300,000, Aldrich), were dispersed into 100 ml deionized water at room temperature. After stirring vigorously for 6 h, the resulting solution was filtered and washed with deionized water several times to remove the remaining ions. The as-obtained deep grey products were dried at 80 °C for 12 h and then transferred to a ceramic crucible and carbonized at 500 °C for 2 h under argon atmosphere. Finally, some grey CNTs/MnO_x_-Carbon powder was obtained. For comparison, some MnO_2_ powder and CNTs/MnO_x_ hybrid nanofibers were also prepared by the liquid chemical redox reaction (LCRR), respectively, similar to that for the CNTs/MnO_x_-Carbon hybrid nanofibers, except that no CNTs and PVP were added during the LCRR process for the MnO_2_ powder and no PVP was added for the CNTs/MnO_**x**_ hybrid nanofibers.

### Materials Characterization

Thermogravimetric analysis (TGA) of the as-prepared CNTs/MnO_x_-Carbon hybrid nanofibers and CNTs/MnO_x_ hybrid nanofibers was carried out with a TGA/DSC1 type instrument (STA449C NETZSCH, German) with a heating rate of 5 °Cmin^−1^ from 35 to 700 °C in air. The phase of the products was examined with an X’ Pert Pro MPD X-ray diffractometer with Cu Kα radiation (λ = 1.5418 Å, Philips, Holland). The morphology of these nanomaterials was evaluated with a NanoSEM 230 field emission scanning electron microscope (FE-SEM, Nova NanoSEM 230, FEI, USA) and a Tecnai G2F20 S-TWIN transmission electron microscope (TEM, Tecnai GX F20 S-TWIN, FEI, USA). The X-ray photoelectron spectroscopy (XPS) experiments were carried out on a VG Scientific ESCALAB 250 instrument (XPS, ESCALAB 250, Thermo Scientific, America) by using aluminum Kα X-ray radiation during XPS analysis.

### Electrochemical Characterization

The electrochemical properties were further measured on electrodes that were prepared by compressing a mixture of the as-prepared active materials (~14 mg, MnO_2_ nanopowders, CNTs/MnO_x_ hybrid nanofibers, or CNTs/MnO_x_-Carbon hybrid nanofibers), carbon black (~3 mg, Super P, MMM, Belgium), and poly (vinyl difluoride) (PVDF) binder (~3 mg) in a weight ratio of 70:15:15, and further uniformly coating the mixture onto ~15 pieces of copper foils (each size:1 cm×1 cm). Pure lithium metal foil was used for the counter and reference electrode. The electrolyte was LiPF_6_ (1 M) in a mixture of ethylene carbonate (EC) and dimethyl carbonate (DMC) (1:1 v/v; MERCK KgaA, Germany). Coin cells were assembled in a high-purity argon-filled glove box. The galvanostatic method was used to measure the electrochemical capacity of the electrodes at room temperature on a LAND-CT2011A instrument with a charge–discharge current density of 100 mAg^−1^ based on the total mass of electrode materials mainly including the active materials, the carbon black and the binder. Rate capability tests of the electrodes were then carried out systematically at various current densities (100 mAg^−1^, 200 mAg^−1^, 500 mAg^−1^, 1000 mAg^−1^). The cut-off potentials for charge and discharge in the cycling and rate tests were all set at 3.0 and 0.01 V versus Li^+^/Li, respectively. Cyclic-voltammetry was performed on a CHI650D electrochemical workstation with the cut-off voltage range between 0.01–3.0 V.

## Additional Information

**How to cite this article**: Yang, Z. *et al.* Facile Synthesis of Coaxial CNTs/MnO_x_-Carbon Hybrid Nanofibers and Their Greatly Enhanced Lithium Storage Performance. *Sci. Rep.* **5**, 17473; doi: 10.1038/srep17473 (2015).

## Supplementary Material

Supplementary Information
