# Supplementary material for: Facile Synthesis of Coaxial CNTs/MnOx-Carbon Hybrid Nanofibers and Their Greatly Enhanced Lithium Storage Performance
Source: Sci Rep. 2015 Dec 1;5:17473. doi: 10.1038/srep17473 (PMC4664925; doi:10.1038/srep17473)
Supplement: Supplementary Information [file srep17473-s1.doc]

Facile Synthesis of Coaxial CNTs/MnO_x_-Carbon Hybrid Nanofibers and Their Greatly Enhanced Lithium Storage Performance

ZunxianYang^1^*, Jun Lv^1^ Haidong Pang^1^, Wenhuan Yan^1^, Kun Qian^1^, Tailiang Guo^1^*, Zaiping Guo^2,3^*

^1^ National & Local United Engineering Laboratory of Flat Panel Display Technology, Fuzhou University, Fuzhou 350002, P. R. China

^2^Institute for Superconducting & Electronic Materials, University of Wollongong, NSW 2522, Australia

^3^School of Mechanical, Materials & Mechatronics Engineering, University of Wollongong, NSW 2522, Australia

Correspondence and requests for materials should be addressed to Zunxian Yang ([yangzunxian@hotmail.com](mailto:yangzunxian@hotmail.com)), zaiping Guo ([zguo@uow.edu.au](mailto:zguo@uow.edu.au)) or Tailiang Guo([gtl_fzu@hotmail.com](mailto:gtl_fzu@hotmail.com))

### Supporting Information

### Captions

Fig. S1 X-ray diffraction pattern of as-prepared pure MnO_2_. (MnO_2_: Tetragonal structure, JCPDS 72-1982).

Fig. S2 FE-SEM image of as-prepared MnO_2_ nanoparticles.

Fig. S3 TGA curves of the as-prepared **(a)** CNTs/MnO_x_ and **(b)** CNTs/MnO_x_-Carbon hybrid nanomaterials. (The TGA results were obtained in air atmosphere.)

Fig. S4  **(a)** Low-magnification TEM image of as-prepared MnO_2_ nanoparticles, **(b)** HRTEM image of MnO_2_ nanoparticles.

Fig. S5 XPS high-resolution spectra of **(a)** the Mn 2p region of the MnO_2_ nanoparticles; **(b)** the O 1s region of the CNTs/MnO_x_ nanoparticles.

Fig. S6 **(a)** Cyclic voltammograms for selected cycles of CNTs/MnO_x_ hybrid nanomaterial electrode from the first cycle to the fifth cycle at a scan rate of 0.1 mVs^-1^ in the voltage range of 0.01-3.0 V; **(b)** Nyquist plots of the MnO_2_, CNTs/MnO_x_ and CNTs/MnO_x_-C composite, respectively.

Scheme S1 Schematic illustration of the formation and charge diffusion mechanism of the CNTs/MnO_x_-Carbon nanofibers:

**(a)** Carbon nanotubes, **(b)** CNTs/MnO_x_ hybrid nanofibers during charge/discharge processes, **(c)** CNTs/MnO_x_-Carbon hybrid nanofibers during charge/discharge processes, **(d)** schematic image of part of a CNTs/MnO_x_-carbon hybrid nanofiber. The inset shows a high-resolution TEM image of the CNTs/MnO_x_-carbon hybrid nanofiber.

**Fig. S1**


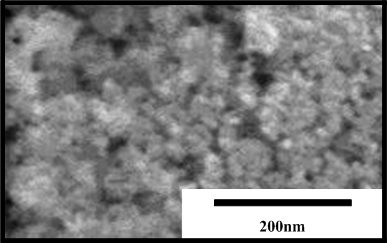


**Fig. S2**

**Fig. S3**


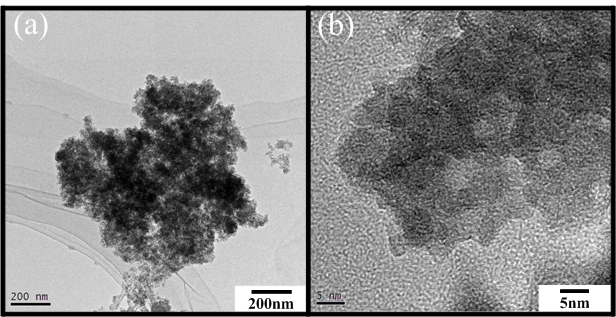


**Fig. S4**

**Fig. S5**

**Fig. S6**


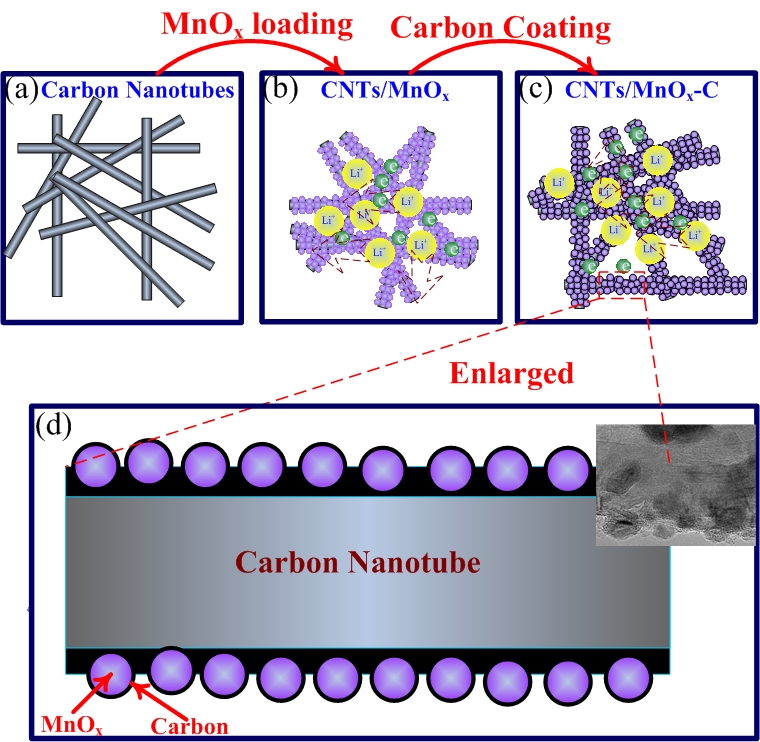


**Scheme S1**
